# On null models for temporal small-worldness in brain dynamics

**DOI:** 10.1162/netn_a_00357

**Published:** 2024-07-01

**Authors:** Aurora Rossi, Samuel Deslauriers-Gauthier, Emanuele Natale

**Affiliations:** Université Côte d’Azur, COATI, INRIA, CNRS, I3S, France; CRONOS, Inria Centre at Université Côte d’Azur, France

**Keywords:** Brain networks, Temporal networks, Small-worldness, Hyperbolic graph, Null model, fMRI

## Abstract

Brain dynamics can be modeled as a temporal brain network starting from the activity of different brain regions in functional magnetic resonance imaging (fMRI) signals. When validating hypotheses about temporal networks, it is important to use an appropriate statistical null model that shares some features with the treated empirical data. The purpose of this work is to contribute to the theory of temporal null models for brain networks by introducing the random temporal hyperbolic (RTH) graph model, an extension of the random hyperbolic (RH) graph, known in the study of complex networks for its ability to reproduce crucial properties of real-world networks. We focus on temporal small-worldness which, in the static case, has been extensively studied in real-world complex networks and has been linked to the ability of brain networks to efficiently exchange information. We compare the RTH graph model with standard null models for temporal networks and show it is the null model that best reproduces the small-worldness of resting brain activity. This ability to reproduce fundamental features of real brain networks, while adding only a single parameter compared with classical models, suggests that the RTH graph model is a promising tool for validating hypotheses about temporal brain networks.

## Author Summary

We show that the random temporal hyperbolic (RTH) graph is a suitable null model for testing hypotheses about brain dynamics, after comparing it with the current state of the art and two other geometric null models. The static version of this theoretical model captures properties of various real-world networks, and its temporal version exhibits the temporal small-world property, for which we propose a new proper temporal definition. In particular, we show that the model best reproduces the temporal small-worldness measured in the empirical temporal network extracted from fMRI signals.

## INTRODUCTION

The brain is always active, even when it is not performing a cognitive task. Therefore, it is interesting to investigate its fluctuations starting from this case: the resting-state condition (Karahanoğlu & Van De Ville, 2017; Maltbie, Yousefi, Zhang, Kashyap, & Keilholz, 2022; Preti, Bolton, & Van De Ville, 2017; Thompson, Brantefors, & Fransson, 2017). In this context, functional magnetic resonance imaging (fMRI) techniques allow us to study the underlying functional architecture of the brain and its temporal evolution. We can represent cerebral activity by extracting and manipulating the blood oxygen level–dependent (BOLD) signal from the scans.

To test certain hypotheses about observed functional connectivity, after modeling brain activity as a complex temporal network (Bahrami, Laurienti, Shappell, Dagenbach, & Simpson, 2022; Sizemore & Bassett, 2018; Thompson et al., 2017), it is important to compare it with an appropriate null model (Lurie et al., 2020; Váša & Mišić, 2022). A null model is a random statistical object that has certain properties in common with the empirical data under consideration and is used to evaluate whether the latter has noteworthy features or properties that cannot be attributed to randomness or other constraints.

In the complex temporal networks of brain activity just mentioned, each node corresponds to a brain region, and edges represent the presence of interactions greater than a certain threshold between two connected regions. As the graph evolves over time, edges may appear and disappear, meaning that the interaction between two brain regions jumps over and below a certain value. Complex networks can be found across different fields such as biology, sociology, epidemiology, and brain dynamics (da Fontoura Costa et al., 2011). They are graphs with nontrivial topological properties; two main properties that have come to define them are scale-freeness and small-worldness (Bassett & Bullmore, 2017; Chung & Lu, 2006; Tomasi, Shokri-Kojori, & Volkow, 2017).

In this paper, the common property of the null model and the real data we focus on is small-worldness, which expresses the efficiency of information exchange between nodes. It is defined as the ratio between two properties of the network: the clustering coefficient and the average path length. The clustering coefficient measures how many connections exist among a node’s neighbors, which reflects the local connectivity of the network. The average path length measures the average number of steps it takes to travel from one node to another, which reflects the global connectivity of the network. Given the fact that we are considering temporal brain networks, the temporal version of small-worldness can be interpreted as a measure of efficient communication among brain regions. We propose a new definition of temporal small-worldness *S* = *C*/*L* as the ratio of the temporal clustering coefficient *C* and the temporal path length *L*. The temporal clustering coefficient *C* is the time average of the clustering coefficients computed at each time step. The temporal path length *L* is the average of the fastest temporal paths between all pairs of nodes. *S* differs from *S*_*SB*_, the temporal small-worldness definition of Sizemore and Bassett (2018), as it replaces the temporal version clustering coefficient with the temporal correlation coefficient *TC*, which measures the change between two consecutive time steps. In the Measures subsection we will discuss the reasons behind the adoption of this new definition.

Until recently, neuroscientists mainly analyzed the static version of brain networks (Bassett & Bullmore, 2017; Fornito, Zalesky, & Bullmore, 2016; Liao, Vasilakos, & He, 2017; Váša & Mišić, 2022), as the temporal extension of graph models is a recent research topic even in computer science. Currently, the temporal null models that have been considered in the literature are the randomized edge model and the randomly permuted times model, which are based on empirical data (Sizemore & Bassett, 2018). For consistency with the other models used in this work, we will refer to them as the random temporal edge (RTE) graph model and the random temporal permuted times (RTPT) graph model. In the RTE graph, each edge is rewired at each time step by changing one of its endpoints in the original graph. In the RTPT graph, the temporal structure is deteriorated by randomly permuting the time at which edges occur. However, as we show in this work, these random models do not reflect the typical structure of small-world networks, which exhibit short average path lengths and low clustering coefficients (Watts & Strogatz, 1998).

Lately, in the research community of real-world complex networks, the random hyperbolic (RH) graph model has become famous because it can exhibit both a high-tail degree distribution and small-worldness (Bode, Fountoulakis, & Muller, 2016; Fountoulakis, 2012; Fountoulakis & Müller, 2016; Krioukov, Papadopoulos, Kitsak, Vahdat, & Boguñá, 2010). It is a geometric model whose nodes are points that lie in the hyperbolic space. The latter cannot be embedded in Euclidean space, owing to the exponential growth of its volume. It also happens in the case of functional brain networks, which are best represented in the hyperbolic disc as shown by Whi, Ha, Kang, and Lee (2022). A temporal version of the RH graph model has recently been considered (Hartle, Papadopoulos, & Krioukov, 2021; von Looz & Meyerhenke, 2018).

We also consider two other temporal geometric graphs, the random temporal square (RTS) graph model and the random temporal torus (RTT) graph model as possible good candidates for null models. We compare them with the state of the art and with the networks extracted from the signals contained in the empirical data of 1,047 subjects from the WU-Minn Human Connectome Project (*WU-Minn HCP 1200 Subjects Data Release Reference Manual*, 2018), which we collected in a dataset that we made publicly available (Rossi, Deslauriers-Gauthier, & Natale, 2023). We test whether our random temporal models have small-worldness values close to those of empirical data at different connectivity thresholds. In particular, we show that the random temporal hyperbolic (RTH) graph model is a suitable null model with respect to the small-worldness property, especially when compared to the previous RTPT and RTE graph null models considered by Sizemore and Bassett (2018).

## METHODS

### Extracting Temporal Brain Networks

In this section, we describe the fMRI preprocessed data we started from and the process we implemented to extract the temporal brain networks from them (Rossi et al., 2023).

#### Data.

The data we used were taken from the Human Connectome Project (*WU-Minn HCP 1200 Subjects Data Release Reference Manual*, 2018), specifically, the 3 Tesla Siemens fMRI dataset of 1,047 selected resting-state subjects (rfMRI) scanned at the University of Washington or the University of Minnesota. The data are preprocessed and minimally preprocessed according, respectively, to Smith et al. (2013) and Glasser et al. (2013), and had artifacts removed using ICA-FIX (Griffanti et al., 2014; Salimi-Khorshidi et al., 2014). During the acquisition of the brain images, the patients would lie quietly in a darkened room with their eyes open, looking at a fixed bright point on a dark background, without performing any tasks. This is arguably the simplest case one can imagine, and it is natural to start the investigation from this point because even when the subject does nothing, brain regions are activated and the signal fluctuates. Understanding this scenario is important to proceed with the comparison between this state and more complicated situations, such as when the patient has some neural disease or is performing cognitive tasks involving memory and attention (Liao et al., 2017).

The rfMRI data were acquired in four runs of 14 min and 33 s each. The data were captured in both right-to-left (RL) and left-to-right (LR) phase encoding, with two scans per direction. We used only one of the two left-to-right scans. Regarding the temporal resolution of the data, the total number of time steps is 1,200 and the interval between two time steps is 0.72 s. Regarding the spatial resolution, there are 91 voxels in the *x*- and *z*-axis and 109 in the *y*-axis, making the total number of voxels 902,629. The dimension of a voxel is 2 × 2 × 2 mm.

#### Processing.

**Figure 1.**
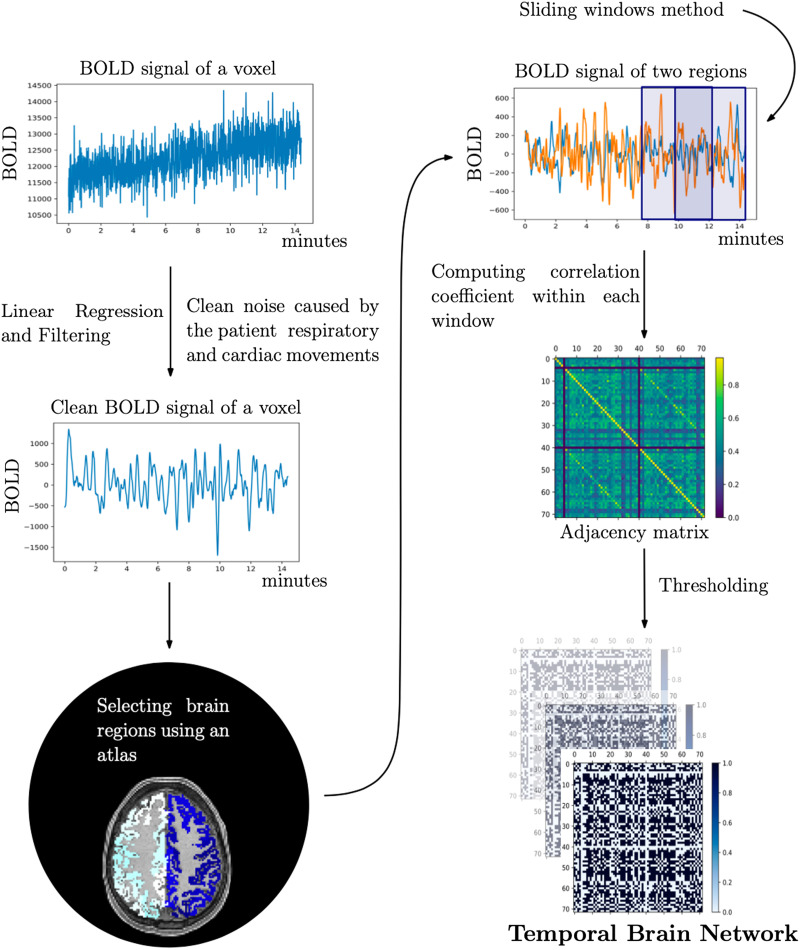
Steps of the pipeline to extract temporal brain networks from an fMRI signal for each voxel of the brain. The first step is linear regression and bandpass filtering of the data. The second step is the division of the brain into regions according to an atlas. The third step is the sliding window method, which individuates rectangular windows within which, in the fourth step, the Pearson correlation coefficient is computed between each pair of brain region time series. The last step is the thresholding of the data.

The pipeline we implemented takes the BOLD signals of the rfMRI scan for each voxel of the brain and transforms them into temporal graphs (Van Dijk et al., 2010). We release them in the publicly available dataset, Rossi et al. (2023). The first step of the pipeline is to perform a linear regression on the movement parameters of the data, which reduces the contribution of the subject’s movements during the 14-min procedure. The data also contain some noise caused by the patient’s involuntary respiratory and cardiac rates. This problem is mitigated by applying a bandpass filter that isolates the BOLD fluctuations within a range of 0.01–0.08 Hz (Van Dijk et al., 2010). To obtain the division of the brain into regions that correspond to the nodes of our temporal networks, we use a brain atlas, such as the Gallardo et al. (2018), Glasser et al. (2016), or Schaefer et al. (2018). Using an atlas, we extract groups of voxels from gray matter areas that differ by anatomical and functional criteria. By averaging their signal, a time series is created for each brain region. Using the sliding window method, we individuate rectangular windows of length 60 s with an overlap of 30 s (Preti et al., 2017), each window being equivalent to one time step of the network. The Pearson correlation coefficient is calculated between each pair of brain region time series within each window; the resulting correlation value is the weight of the edge between the respective brain region nodes. In this way, we obtain an adjacency matrix for each window. The rows and columns of the matrix are ordered so that regions of the left hemisphere are grouped in the first block and regions of the right hemisphere are grouped in the second block (see the adjacency matrix in Figure 1). The two more correlated blocks (corresponding to the lighter pixels in the figure), upper left and lower right, represent strong intrahemispheric connectivity, while the two extra-diagonal blocks correspond to weaker interhemispheric connections. Three diagonals can be distinguished, the middle one representing the correlation of a region with itself. The other two sub-diagonals correspond to the connectivity of each region with its counterpart in the opposite hemisphere (Fornito et al., 2016). The values range between –1 and 1. If they are high (close to 1), the interpretation is clear: The two areas are synchronously activated, thus more likely to communicate. On the other hand, there is no consensus on the interpretation of negative indices. Given that many measures are defined for unweighted graphs, it is also common to threshold the data (Fornito et al., 2016). Currently, there is no consensus on the most appropriate threshold, so we analyze the data according to different positive thresholds applied to the Pearson correlation coefficient. The sequence of the obtained matrices defines the temporal brain networks.

### Random Graph Models

In this paper, we consider five temporal random graph models. The first two models are the random temporal permuted times (RTPT) graph model and the random temporal edges (RTE) graph model (Sizemore & Bassett, 2018), which are constructed by randomizing empirical data; in particular, the RTPT graph is obtained by randomizing the time at which each edge appears, while the RTE graph is obtained by randomly reassigning an edge endpoint to a different node within each time step. The other three models are the random temporal square (RTS) graph model, the random temporal torus (RTT) graph model, and the random temporal hyperbolic (RTH) graph model, which do not depend on empirical data. They are synthetic graphs obtained by randomly placing nodes on different spaces: the square, the torus, and the hyperboloid, respectively. At each time step, the temporal graph is constructed by connecting the nodes depending on their position. From one time step to the next, the node positions are updated according to a movement model. Both the adjacency and their movement are computed according to the geometry of the underlying space. A summary of the five models and their parameters can be found in Table 1. Next, we provide their formal mathematical definitions. In the next section, we will compare them with the temporal brain networks extracted from data.

**Table 1.** Summary of the random temporal graph models described in the Random Graph Models section.

Name	Acronym	Data-based or synthetic	Topological space	Parameters	References
*Random temporal permuted times*	RTPT	Data-based	–	𝒢	Sizemore and Bassett (2018)
*Random temporal edges*	RTE	Data-based	–	𝒢	Sizemore and Bassett (2018)
*Random temporal square*	RTS	Synthetic	Square [0, 1]^2^	*r*, *v*	Penrose (2003)
*Random temporal torus*	RTT	Synthetic	Torus [0, 1]^2^	*r*, *v*	Penrose (2003)
*Random temporal hyperbolic*	RTH	Synthetic	Hyperboloid with *ζ* < 0	*ζ*, *α*, *R*, *v*	Krioukov et al. (2010), von Looz and Meyerhenke (2018)

#### Temporal graph.

A temporal graph, denoted as *G*(*V*, *E*_*T*_), is given by a set of nodes *V* and a set of temporal edges *E*_*T*_. Each temporal edge is a triple (*u*, *v*, *t*) where *u*, *v* ∈ *V* are the endpoints of the edge and *t* ∈ [1, …, *T*] is the time at which the edge appears thus *E*_*T*_ = {(*u*, *v*, *t*) : *u*, *v* ∈ *V*, *t* ∈ [1, …, *T*]}.

#### Random temporal permuted times graph model (RTPT(**G**)).

**Figure 2.**
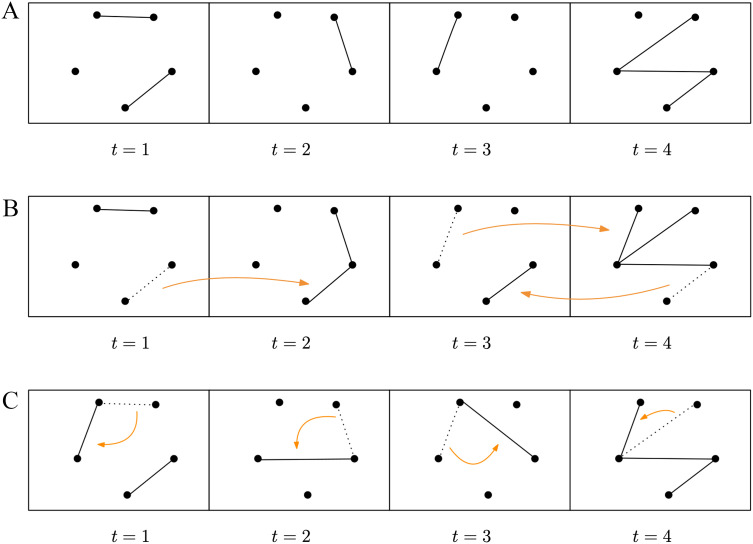
Example of 4-time steps illustrating how the RTPT and RTE graphs are obtained from a given temporal graph (A). The RTPT graph (B) is obtained by randomizing the time each edge appears over different time steps. The RTE graph (C) is obtained by reassigning one endpoint of an edge to a different node within each window time step.

The random temporal permuted times graph model (Sizemore & Bassett, 2018) is obtained by taking the temporal graph *G*(*V*, *E*_*T*_) of a subject (see Figure 2A) and randomizing the time at which each edge appears (see Figure 2B). We construct a *G*(*V*, $E_{T}^{\prime }$) from RTPT(*G*) model by sampling a permutation *π* uniformly at random and defining the set $E_{T}^{\prime }$ = {(*u*, *v*, *π*(*t*)) : (*u*, *v*, *t*) ∈ *E*_*T*_}.

#### Random temporal edges graph model (RTE(**G**)).

The random temporal edges graph model (Sizemore & Bassett, 2018) is obtained from the temporal graph *G*(*V*, *E*_*T*_) of a subject (see Figure 2A) by reassigning one endpoint of an edge to another node in each window time step (see Figure 2C). We construct a *G*(*V*, $E_{T}^{\prime }$) from RTE(*G*) by sampling a permutation *σ* uniformly at random of the set *V* and by setting (*u*, *σ*(*v*), *t*) ∈ *E*′ for each contact (*u*, *v*, *t*) ∈ *E*.

#### Random square graph model (RS(*r*)).

**Figure 3.**
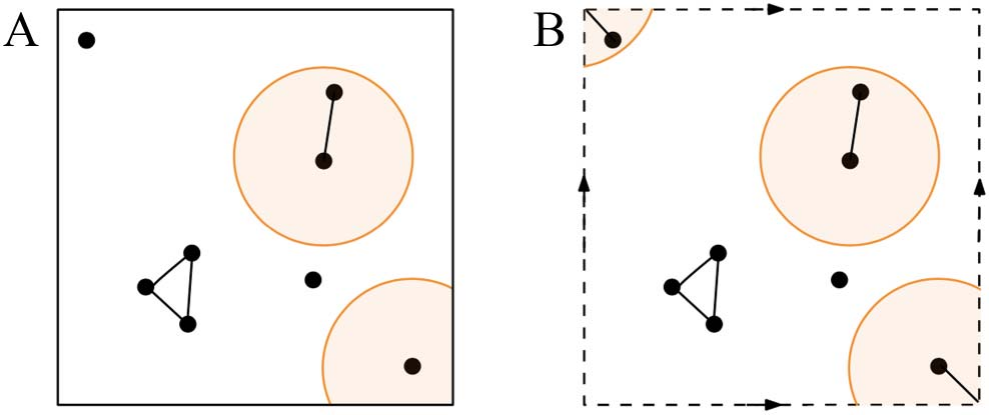
Showing the main difference between the RS (A) and the RT (B) graphs. In both cases, the nodes are connected if they are at a distance less than a radius *r* In the RS graph the nodes are distributed on a square, hence the node on the bottom right and the node on the top left are not connected; on the torus, on the contrary, they are connected.

An RS(*r*) graph *G*(*V*, *E*) (Penrose, 2003) is obtained by distributing nodes independently and uniformly at random on a unit square [0, 1]^2^ and connecting each pair of nodes whose Euclidean distance is less than radius *r* (see nodes contained in orange balls in Figure 3A). The set of edges is thus *E* = {(*u*, *v*) : (*u*, *v*) ∈ *V* × *V*, ‖*p*_*u*_ − *p*_*v*_‖_2_ ≤ *r*}, where *p*_*u*_ and *p*_*v*_ are the coordinates of the positions of the points *u* and *v*.

#### Random torus graph model (RT(*r*)).

A RT(*r*) graph *G*(*V*, *E*) is obtained by distributing nodes independently and uniformly at random on a torus [0, 1]^2^ and connecting each pair of nodes that are at a distance less than radius *r* (see nodes contained in orange balls in Figure 3B). On the torus [0, 1]^2^, the distance between two nodes *p*_*A*_, *p*_*B*_ with coordinates, respectively (*x*_*A*_, *y*_*A*_), (*x*_*B*_, *y*_*B*_), is defined as

$$d\left(p_{A},p_{B}\right)=\sqrt{{\left(\frac{1}{2}-\left|\frac{1}{2}-\left|x_{A}-x_{B}\right|\right|\right)}^{2}+{\left(\frac{1}{2}-\left|\frac{1}{2}-\left|y_{A}-y_{B}\right|\right|\right)}^{2}}.$$

#### Temporal version of random square and torus models.

To obtain the temporal version of both the RS and RT graph models, which we will refer to them as RTS(*r*, *v*) and RTT(*r*, *v*) graph models, we can define a movement model according to which the positions of the points are updated and the distances are recomputed. The number of updates is set equal to the number of time steps of the real data. The new position of a point is determined by applying a displacement vector to it, whose direction is chosen uniformly in [0, 2*π*) and whose length is chosen uniformly in (0, *v*). The *v* is called the speed parameter. In the case of an RT graph, there is no problem of updating the point position, since there are no boundaries. In the case of the unit square, boundaries need to be taken into account. If a point happens to move outside the boundary of the unit square, its trajectory is reflected inwards, meaning that its position is updated as if it had bounced off the boundary.

#### Random hyperbolic graph model (RH(*ζ*, *α*, *R*)).

**Figure 4.**
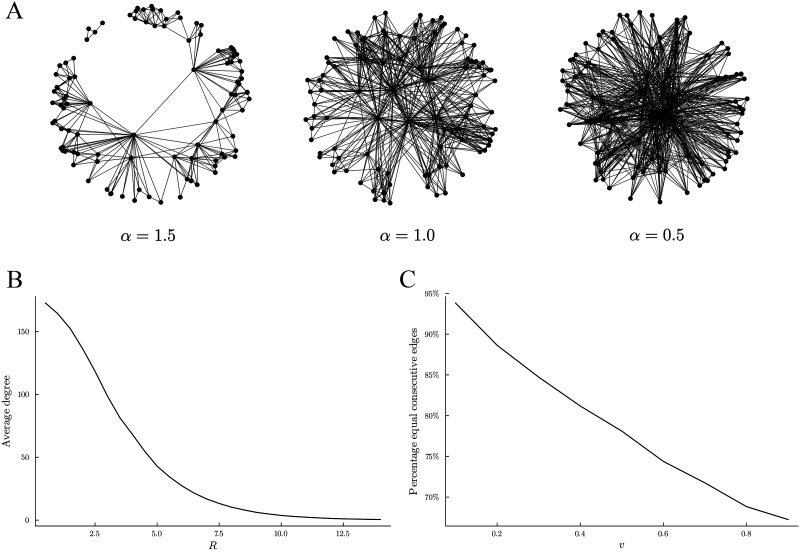
Behavior of the RTH graph model parameters. (A) Representations of the RTH graph on the Poincaré disk for different values of the parameter *α*. (B) Example showing how the parameter *R* affects the average degree of the RTH graph when the other parameters are fixed *α* = 1.2, *ζ* = 1, and *v* = 0.9. (C) Example showing how the parameter *v* affects the percentage of edges that remain the same between two consecutive time steps when the other parameters are fixed *α* = 0.5, *R* = 4.5, and *ζ* = 1.

Krioukov’s method for generating a random hyperbolic graph is to distribute nodes quasi-uniformly within a disk of radius *R* centered on the upper half of a hyperboloid of given negative curvature *K* = −*ζ*^2^ (Krioukov et al., 2010). Writing the node positions in polar coordinates (*r*, *θ*), where *r* ∈ [0, *R*] and *θ* ∈ [0, 2*π*], we have that the angular density is *ρ*(*θ*) = $\frac{1}{2\pi }$ and the radial coordinate density is *ρ*(*r*) = *α*$\frac{\sinh\left(\alpha r\right)}{\cosh\left(\alpha R\right)-1}$. The *α* parameter controls the spread of the point positions. If *α* = *ζ*, the points follow a uniform distribution. If *α* > *ζ*, the points are more likely to be near the border of the disk; otherwise, they are more likely to be near the center (see Figure 4A). Two nodes *u* and *v* are connected if they are at a hyperbolic distance *d*_*H*_ less than *R*, where, considering the polar coordinate of *u* = (*r*_*u*_, *θ*_*u*_) and *v* = (*r*_*v*_, *θ*_*v*_), *d*_*H*_ is defined as

$$d_{H}\left(\left(r_{u},\theta_{u}\right),\left(r_{v},\theta_{v}\right)\right)=\frac{1}{\zeta }\text{acosh}\left(\cosh\zeta r_{u}\cosh\zeta r_{v}-\sinh\zeta r_{u}\sinh\zeta r_{v}\cos\left(\pi -\left|\pi -\left|\theta_{u}-\theta_{v}\right|\right|\right)\right).$$

Thus, the parameter *R* affects the average degree of the graph. The higher the value of *R*, the larger the distances between the nodes and the lower the average degree (see Figure 4B). As for the curvature parameter *ζ*, we set it to *ζ* = 1, since (Lemma 1.1 of Bode et al., 2016) if two RH graphs have the same ratio $\frac{\zeta }{\alpha }$ and the other parameters are equal, then they produce the same distribution on graphs.

To obtain a temporal version, which we will refer to it as the RTH(*ζ*, *α*, *R*, *v*) graph model, we update the point position as many times as the number of time steps of the real data we want to compare with. The point position update is chosen in such a way that guarantees that the marginal distribution of each time step is the same as the initial one. Specifically, the polar coordinate is updated by adding to the previous value a number chosen according to a uniform distribution over (0, *v*), and then computing the modulo 2*π*. For the radial coordinate, we add to the previous value a number chosen according to a uniform distribution over (−*v*, *v*), and we reflect the result in the interval [0, 1] to keep the distribution uniform in [0, 1] (Hartle et al., 2021). Thus, this parameter controls the movement of the points, and the higher the value of *v*, the more the points move and the lower the percentage of edges that remain the same between two consecutive time steps (see Figure 4C).

### Measures

In this section, we introduce some measures to define a temporal version of small-worldness. The first static qualitative definition of small-worldness was introduced by Watts and Strogatz (1998) as the combination of a high clustering coefficient, as in lattice graphs, and a short average path length, as in random graphs. Humphries and Gurney (2008) combined the previous measures into a quantifiable ratio: clustering coefficient divided by average path length, normalized by the corresponding measure calculated on a random graph, formally $\frac{C_{g}}{L_{g}}/\frac{C_{\mathrm{rand}}}{L_{\mathrm{rand}}}$. If the ratio is greater than 1, it indicates a significant small-world property of the network. The extension to temporal complex networks requires adapting the previous measures to capture the time evolution. The path length can naturally be extended to the average length of the fastest time-respecting path between each pair of nodes. Tang, Scellato, Musolesi, Mascolo, and Latora (2010) replaced the local clustering coefficient with the temporal correlation coefficient, which was also considered by Sizemore and Bassett (2018), which we will define in the next paragraph.

#### Temporal clustering coefficient.

**Figure 5.**
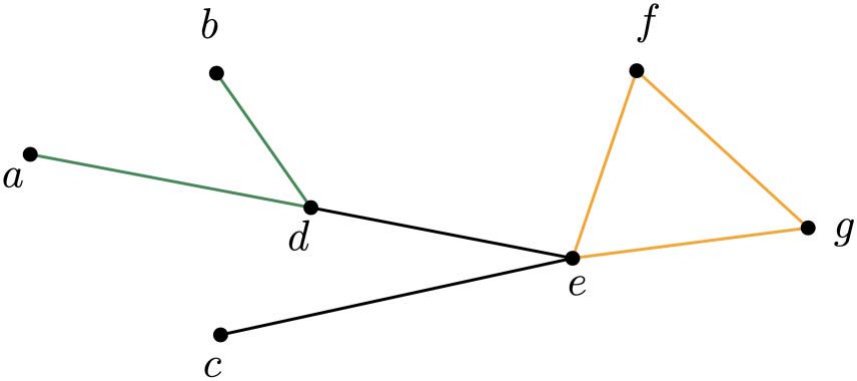
Open and closed triplets. The open triplet in this graph is the green path; instead, the orange triangle is a closed triplet.

The clustering coefficient is defined as the ratio between the total number of closed triplets and the total number of triplets (open and closed). More specifically, closed triplets are sub-triangles in the graph that are counted three times, and open triplets are paths of length two, as illustrated in Figure 5.

The clustering coefficient at a given time *t* is denoted by *C*(*t*) and is required to compute the temporal clustering coefficient.

$$C\left(t\right)=\frac{\sum_{i,j,k}A_{ij}\left(t\right)A_{jk}\left(t\right)A_{ki}\left(t\right)}{\sum_{i}k_{i}\left(k_{i}-1\right)}$$ (1)

where *k*_*i*_ = ∑_*j*_ *A*_*ij*_(*t*) and *A*_*ij*_(*t*) is the value of the binary adjacency tensor of the network at time *t* corresponding to nodes *i* and *j*. Formula 1 is equivalent to the one introduced by Watts and Strogatz (1998): The clustering coefficient is the average over all nodes of the local clustering coefficient

$$C_{i}=\frac{2|\left\{e_{jk}:v_{j},v_{k}\in N_{i},e_{jk}\in E\right\}|}{k_{i}\left(k_{i}-1\right)},$$

**Figure 6.**
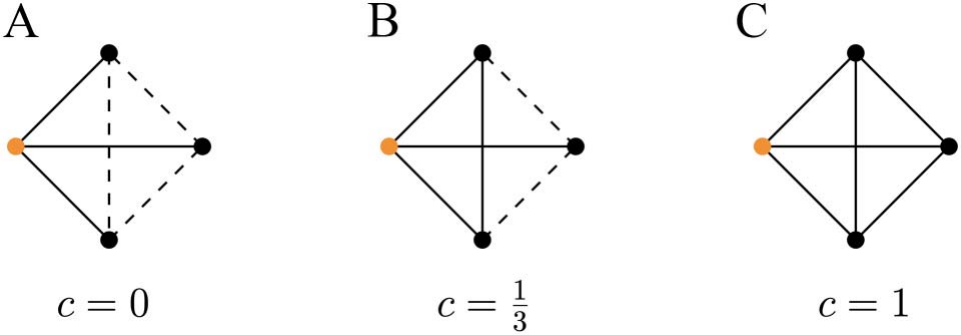
Local clustering of the orange node. In (A) the clustering coefficient is equal to 0 because there are zero connections among the neighbors of the orange node, over three possible ones. In (B) there is one connection over three. In (C) there are all possible connections, hence the clustering coefficient has value 1, which is the maximum.

*C*_*i*_ measures the tendency of neighbors of a node to form a clique. Some examples are shown in Figure 6.

The temporal clustering coefficient *C* is the average of the clustering coefficient computed at each time step:

$$C=\frac{1}{T}\sum_{t}C\left(t\right).$$

#### Temporal path length.

The temporal path length is defined as the average of the fastest paths between all pairs of nodes. Paths are taken across time. Only one edge can be used at each time step, and pauses are allowed at nodes (i.e., an edge can be not selected to move forward in a time step). The temporal path length *L* is thus defined as follows:

$$L=\frac{1}{N\left(N-1\right)}\sum_{i\neq j}l_{ij},$$

where *l*_*ij*_ is the minimum time needed to get from *i* to *j* starting at time 0. If there is no path between two nodes, the distance is set to the maximum distance, which is the number of time steps. Fornito et al. (2016) suggest other ways to address the latter case, such as considering only paths that exist or choosing a threshold that yields only connected graphs.

#### Temporal correlation coefficient.

The temporal correlation coefficient quantifies how much the temporal structure changes over time. In particular, it shows the tendency of a node’s neighbors to remain connected to it over successive time steps. The higher the coefficient, the smaller the changes over time (Sizemore & Bassett, 2018). The temporal correlation coefficient of a node *i* is defined as

$${TC}_{i}=\frac{1}{T-1}\sum_{t=1}^{T-1}\frac{\sum_{j}A_{ij}\left(t\right)A_{ij}\left(t+1\right)}{\sqrt{\sum_{j}A_{ij}\left(t\right)\sum_{j}A_{ij}\left(t+1\right)}}.$$

We can thus write the temporal correlation coefficient for the whole temporal network as

$$TC=\frac{1}{N}\sum_{i}{TC}_{i}.$$

#### Temporal small-worldness.

**Figure 7.**
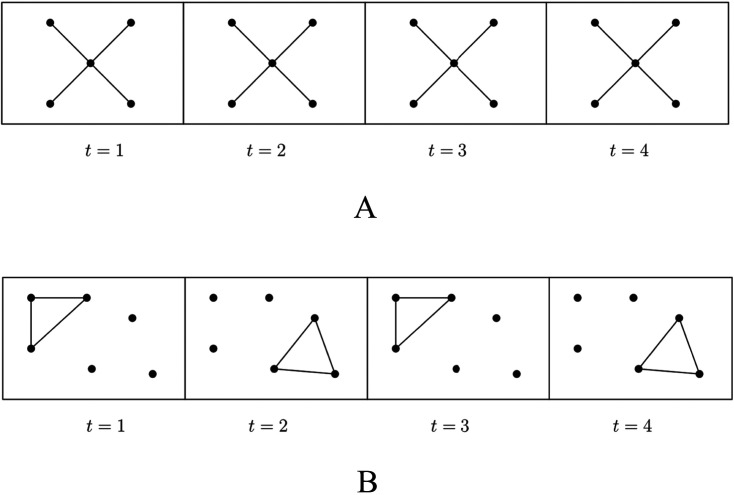
Small examples of the temporal graphs where the temporal correlation coefficient differs from the temporal clustering coefficient in a significant way. (A) A five-node star graph. Its temporal correlation coefficient is 1, while its temporal clustering coefficient is 0. (B) A six-node temporal graph. Its temporal correlation coefficient is 0, while its temporal clustering coefficient is 0.5.

In the static case, which corresponds to a graph that remains unchanged at each time step, the average path length is retrieved from the temporal path length. On the contrary, in the latter case, the temporal correlation coefficient does not coincide with the clustering coefficient. For this reason, the temporal correlation coefficient does not seem to properly generalize the clustering coefficient by Watts and Strogatz (1998) to the temporal setting, while our definition appears to be consistent with the latter. To illustrate the differences between the two measures, consider the following simple examples of temporal graphs with four time steps. In the first, shown in Figure 7A, we have a five-node star graph that does not change over time in which the temporal correlation coefficient is 1, while the temporal clustering coefficient is 0 because there are no closed triplets. In the second case, we have a six-node temporal graph in which a three-node triangle subgraph alternates between two configurations, as shown in Figure 7B. In this case, the temporal correlation coefficient is 0, while the temporal clustering coefficient is 0.5. As a consequence, in our experiments, we consider and compare two versions of small-worldness:

- The *temporal small-worldness* is the ratio between the clustering coefficient and the temporal path length, without normalization; formally,
  $$S=\frac{C}{L}.$$
- The *Sizemore and Bassett temporal small-worldness* is the ratio between the temporal correlation coefficient and the temporal path length, without normalization; in formulas,
  $$S_{SB}=\frac{TC}{L}.$$

## EXPERIMENT SETUP

In this section, we provide some technical information, such as the details of the machine on which we run the experiments and the inputs of the pipeline, and we describe the optimization methods we used to choose the model parameters.

### Computing Platform

We run the pipeline on the Nef Cluster Computing Platform of Inria Sophia Antipolis. By setting the parameters described below, the average execution time of our pipeline for a subject is 250 s, on a 64-core node with the following technical characteristics:

- AMD dual-EPYC 7542 processors,
- 1024 GB of RAM.

### Pipeline

The pipeline is implemented in Python. As explained in the Methods section, it transforms the BOLD signal into a temporal graph. The code, which is available at https://github.com/aurorarossi/TemporalBrainNetworksCode (Rossi, 2024), takes various parameters as input, which are described in the next sections.

#### Atlas.

Regarding the type of atlas, it is possible to choose among Gallardo et al. (2018), Schaefer et al. (2018), or Glasser et al. (2016). For the first two, it is possible to select the number of regions into which the brain is divided, starting from 102 nodes up to a maximum of 1,002 nodes. The Glasser atlas instead has 362 regions. In our experiment, we chose the Schaefer atlas with 302 nodes. Using an atlas with a higher number of regions could potentially introduce errors due to the small size of the regions, which could result in regions not matching across different subjects.

#### Windows.

Several parameters describe the windows, such as the seconds corresponding to the window length and the window overlap, which we chose to be 60 and 30 s, respectively. Among the different types of windows, we chose to work with rectangular windows.

#### Correlation.

As correlation measure we chose the classical Pearson’s correlation.

#### Thresholds.

Since there was no consensus on the most appropriate threshold, we consider different values. Specifically, we consider all the thresholds from 0.2 to 0.9 with a step of 0.05 and from 0.9 to 0.98 with a step of 0.02. The output is a binary temporal network. The latter is sparse for high threshold values and dense for low values.

### Generators and Measures

All temporal graph generators and measures are written in the Julia programming language. In addition to optimizing the execution of serial code, we applied parallel programming techniques such as multithreading, which were exploited to make the scripts efficient. These scripts take as input 532 GB of data generated by the pipeline starting from 975 GB of raw data.

### Optimization: Fitting Parameters

After creating the temporal graphs with the pipeline, our goal is to compare them with the random temporal models, which all depend on some parameters. The latter should be chosen to share as much as possible some common properties with the real data. In particular, we aim to have the same number of nodes, average degree, and similar temporal small-worldness values. We thus choose the set of parameters for which the random temporal models best match the aforementioned properties in the empirical data. In the RTPT and RTE graph models, the number of nodes and the average degree are inherited from the real data by construction and they do not depend on other parameters. For the RTS and RTT graph models, the number of nodes can be set directly and, by changing the connection parameter *r*, it is possible to obtain any average degree; instead, the velocity parameter *v* has to be optimized. For the RTH graph model, the number of nodes has to be set, the average degree is obtained by changing *R*, and the parameters to be optimized are *α* and *v*, while *ζ* is set to 1, because, as explained in the Methods section, we can fix *ζ* and vary just *α*. We used two different optimization methods to find the best values of the parameters.

**Figure 8.**
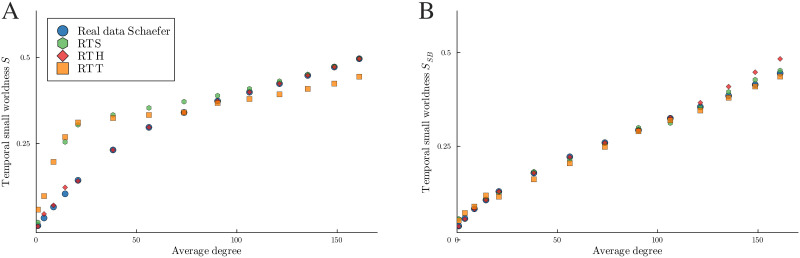
Results of the first optimization method: Comparison between empirical and random networks with 302 nodes at each average degree for the two definitions of small-worldness.

The first finds a set of parameters for the three random temporal models that would fit the small-worldness values of the real data for some average degree values in the interval 0–170 (see Figure 8). To obtain different average degrees for the real data networks we use different threshold values; the higher the threshold value, the lower the average degree. For the other models, we mention how to set it in the previous paragraph.

**Figure 9.**
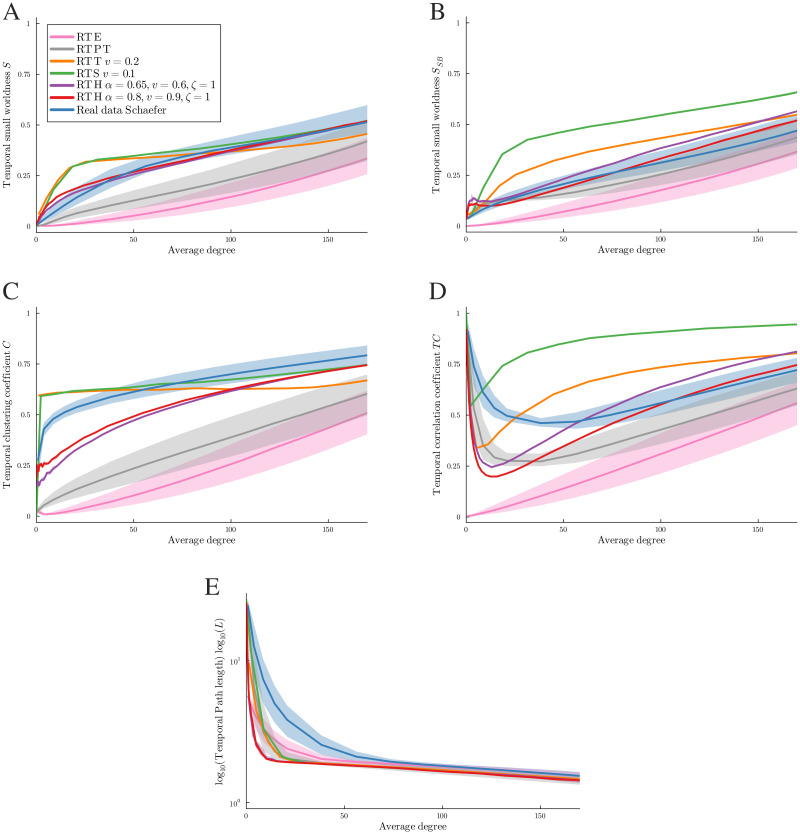
Results of the second optimization method: Comparison betweem empirical and random networks with 302 nodes for different measures.

The second optimization method is to select a set of parameters for each random model that minimizes the area between the temporal small-worldness curves of the empirical data and the random temporal model over all different average degrees. The curves are obtained by linear interpolation of the temporal small-worldness values at different average degrees (see Figure 9). For the RTH graph model, two different sets of parameters are shown, one optimizing each definition of temporal small-worldness. For the RTS and RTT graph models, only one set of parameters is shown, corresponding to the result of optimization with respect to our definition of temporal small-worldness.

## RESULTS

This section discusses the result of all the temporal measures described in this work, applied to the processed data from the Human Connectome Project (*WU-Minn HCP 1200 Subjects Data Release Reference Manual*, 2018) and to the random temporal models. We have calculated the values over different average degrees, namely in the range 0–170. Those of the empirical data vary by changing the threshold values. Consequently, this happens also for the RTPT and RTE graph models. As for the RTS and RTT graph models, this property depends on the radius of the connection: The higher the radius, the higher the average degree. In the hyperbolic case, as shown in the section above, it depends on *R*, which is the connection radius.

The plots in Figure 8 show the results of the first optimization method. The real data of all 1,047 patients are shown in blue, the RTH graph model in red, and the RTT and RTS graph models in orange and green, respectively. Figure 8A shows that for any average degree value we can find a set of parameters for the RTH graph model that can approximate the real data value of our definition of temporal small-worldness, while this is not the case for RTT and RTS graph models. Figure 8B shows that for any average degree value we can find a set of parameters for all the random models that can approximate the real data value of the temporal small-worldness value of Sizemore and Bassett (2018).

In the plots of Figure 9, as above, the real data are shown in blue. RTT and RTS graph models are shown in orange and green, respectively. RTPT and RTE graph models are shown in gray and pink, respectively. There are also two RTH graph models with different parameters. The one with *α* = 0.65, *v* = 0.6, and *ζ* = 1, illustrated in violet, better matches the small-worldness *S* value of the real data, while the one with *α* = 0.8, v = 0.9, and *ζ* = 1, illustrated in red, better fits the definition of small-worldness *S*_*SB*_, which is based on the temporal correlation coefficient *TC*. For all models, the lines are the medians and the shadows correspond to the values between the first and third quartiles. Looking at the random models, the median of the RTPT graph model always has higher values with respect to those of the RTE graph model for all measures except the temporal path length (see Figure 9E). Note that for RTT, RTS, and RTH graph models the shadows are barely visible because the variance is very small. The quantitative results describing the plots 9A and 9B are given in Table 2 and Table 3, respectively. These tables illustrate the comparison between the median of the empirical data and that of the models. This comparison is made in two ways: by measuring the area between the curves, as explained in the Optimization subsection, and by using different types of norms, namely minimum, maximum, mean absolute, and mean Euclidean distance norms. The latter are computed across the differences between the linear interpolation of the empirical data and the model data values of *S* and *S*_*SB*_. These differences are computed on 16,861 equispaced points in the range [0, 170]. The minimum captures the smallest difference between the curves, while the maximum captures the largest. The mean absolute value norm considers the absolute value of all differences at the selected points and then calculates their average. Analogously, the mean Euclidean distance norm calculates the Euclidean distance between the curves at the selected points and then calculates their average. The best results are highlighted in bold and the second best are underlined. In Table 2 we can see that the RTH graph model with *α* = 0.65, *v* = 0.6 is the one that best approximates the small-worldness *S* value of the real data, and the second best one is the other RTH graph model. In Table 3 we can see that the RTH graph model with *α* = 0.8, *v* = 0.9 is the one that best approximates the small-worldness *S*_*SB*_ value of the real data, while the second best one is the RTPT graph model.

**Table 2.** Distances between the median of the empirical data and the one of the model for the temporal small-worldness *S*, as discussed in the Results section. The best results are highlighted in **bold** and the second best is underlined.

Model	Area	Minimum	Maximum	Mean absolute value	Mean Euclidean distance
RTH *α* = 0.8, *v* = 0.9	2.84	0.0000014	0.067	0.017	0.00018
RTH *α* = 0.65, *v* = 0.6	**2.73**	**0.0000011**	**0.040**	**0.016**	**0.00015**
RTS *v* = 0.1	8.02	0.0052702	0.166	0.048	0.00053
RTT *v* = 0.2	9.88	0.0000023	0.169	0.059	0.00057
RTE	33.92	0.0115314	0.248	0.201	0.00160
RTPT	21.42	0.0128656	0.166	0.127	0.00102

**Table 3.** Distances between the median of the empirical data and the one of the model for the temporal small-worldness *S*_*SB*_, as discussed in the Results section. The best results are highlighted in **bold** and the second best results are underlined.

Model	Area	Minimum	Maximum	Mean absolute value	Mean Euclidean distance
RTH *α* = 0.8, *v* = 0.9	**4.46**	**0.0000006**	0.067	**0.026**	**0.00024**
RTH *α* = 0.65, *v* = 0.6	9.60	0.0093862	0.096	0.057	0.00050
RTS *v* = 0.1	37.23	0.0000018	0.266	0.221	0.00174
RTT *v* = 0.2	17.91	0.0136169	0.130	0.106	0.00084
RTE	20.56	0.0396405	0.142	0.122	0.00095
RTPT	7.25	0.0000087	**0.060**	0.043	0.00035

In all plots, the situation is more noisy in the sparse regime. For the sake of completeness, we did not remove this part even though these regimes are rarely considered in the literature in neuroscience.

## DISCUSSION AND CONCLUSIONS

The main focus of this work is to investigate a novel appropriate null model for testing hypotheses about brain dynamics. A good null model must have similar properties to the empirical data under consideration. In this context, the two most important properties present in complex networks such as those modeling brain activity are small-worldness and scale-freeness. In this work, we focus on the small-worldness property.

To transform the data into temporal networks, the empirical data are collected in time and processed using the sliding window method. The main challenge is to adapt all definitions of measures and models to their temporal versions. In particular, we provide a new definition of temporal small-worldness that is consistent with its static version: If the time steps of the temporal network are all identical, the temporal small-worldness is equal to the static small-worldness.

We compare five different temporal models by looking at how close their temporal small-worldness values are to the ones of the data at different thresholds. In Figure 8 we compare the model at each threshold individually, while in Figure 9 we compare them across different thresholds. The two models introduced by Sizemore and Bassett (2018) perform worse than the other three models for the *S* measure. This may be not surprising given the fact that they do not have any parameters to optimize but depend only on the data. The RTS and RTT graph models perform better than the other two models, and the measures computed on them are consistently very close to each other. This similarity may be explained by the fact that the two graphs look locally the same for all points that are not close to the border of the square or the torus, since the connectivity only differs in the latter area (see Figure 3). The RTH graph model is the one that not only achieves the closest values of the temporal small-worldness, but its curve of values follows the same trend as that of empirical data across different average degrees. This result is consistent with the fact that RTH is a popular complex network model that exhibits both small-worldness and scale-freeness.

This analysis could be similarly extended to the more challenging investigation of a temporal version of the scale-freeness property. It would be interesting to see whether the RTH graph model is still preferable. In this work, we focus on the resting-state fMRI in healthy patients. A further development could be the comparison with patients with diseases that affect the functionality of the whole brain. For example, we could consider diseases such as Alzheimer’s and Parkinson’s but also ADHD, depression, and schizophrenia. Again, the main question is whether the best model to fit the data remains the same across different parameter ranges. Another condition that could be investigated is the case of nonresting state, where patients are engaged in cognitive processes generated by tasks involving memory, learning, and attention. On the null models side, as future work, one could consider the case in which one starts from phase randomization and autoregressive randomization null data of brain dynamics, transforms them in temporal networks, and then applies the same analysis as in this work. This would allow a comparison of the results of the two different approaches (Liégeois, Laumann, Snyder, Zhou, & Yeo, 2017; Liégeois, Yeo, & Van De Ville, 2021).

## ACKNOWLEDGMENTS

This work has been supported by the French government, through the UCA DS4H Investments in the Future project managed by the National Research Agency (ANR) with the reference number ANR-17-EURE-0004. The authors are grateful to the OPAL infrastructure from Université Côte d’Azur for providing resources and support.

## AUTHOR CONTRIBUTIONS

Aurora Rossi: Data curation; Formal analysis; Investigation; Methodology; Software; Validation; Visualization; Writing – original draft; Writing – review & editing. Samuel Deslauriers-Gauthier: Data curation; Formal analysis; Investigation; Methodology; Supervision; Validation; Writing – review & editing. Emanuele Natale: Conceptualization; Formal analysis; Funding acquisition; Investigation; Methodology; Project administration; Resources; Supervision; Writing – original draft; Writing – review & editing.

## FUNDING INFORMATION

Aurora Rossi, Agence Nationale de la Recherche (https://dx.doi.org/10.13039/501100001665), Award ID: ANR-17-EURE-0004.

## TECHNICAL TERMS

Null model: A theoretical model used to test hypotheses about empirical data, sharing certain properties with them.
Threshold: A criterion value that determines whether values above it are taken into account and values below it are not.
Node degree: Number of edges incident to a node.
High-tail distribution: A distribution that presents a significant number of extreme events.
Temporal graph: A graph in which edges and nodes change over time. It can be represented as a sequence of graphs, each corresponding to a time step of the network at a given time.
Brain atlas: Subdivision of the gray matter of the brain into smaller areas that share a common property.
Adjacency matrix: A square matrix representing the graph structure, where the indices of rows and columns represent the nodes and the entries correspond to the edge weight between the respective nodes.
Lattice graph: A graph that contains a regular pattern.
Time-respecting path: A finite or infinite sequence of distinct edges, ordered in time, connecting a set of distinct nodes.
Binary temporal graph: A temporal graph whose edges have weights of 1 or 0.

## Supporting Information

https://github.com/aurorarossi/TemporalBrainNetworksCode
